# Supplementary material for: Stress experience and hormone feedback tune distinct components of hypothalamic CRH neuron activity
Source: Nat Commun. 2019 Dec 13;10:5696. doi: 10.1038/s41467-019-13639-8 (PMC6911111; doi:10.1038/s41467-019-13639-8)
Supplement: Supplementary file 1 — Supplementary Information [file 41467_2019_13639_MOESM1_ESM.pdf]

Supplementary Information

Stress experience and hormone feedback tune distinct components of hypothalamic CRH neuron activity

Supplementary Information

Stress experience and hormone feedback tune distinct components of hypothalamic CRH neuron activity

Supplementary Figure 1

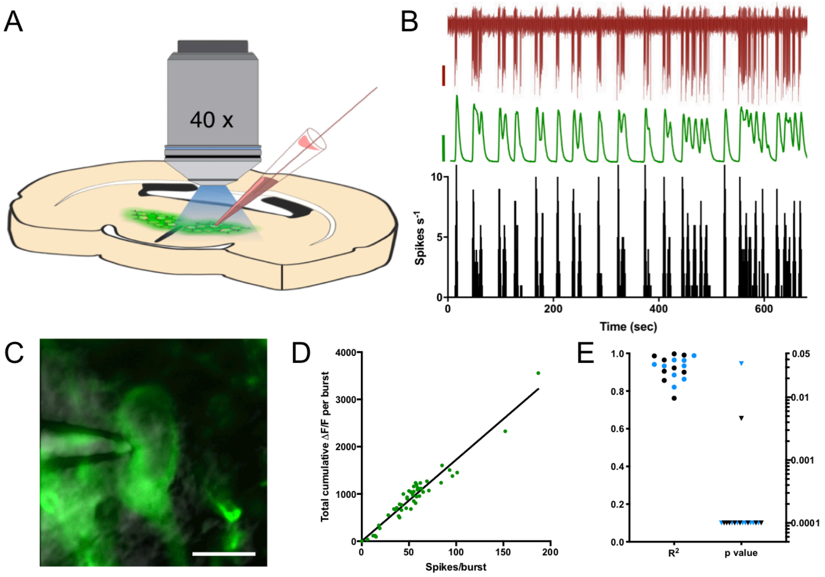

Supplementary Figure 1. GCaMP6s reliably reports CRH neuron spike output.

A) Schematic illustration of dual GCaMP6s imaging and loose-patch electrophysiology in an acute brain slice. B) Representative traces (top, red; scale bar: 50pA) and ratemeter (bottom, black) of loose patch recording with simultaneous GCaMP6s imaging (middle, green; scale bar: 100% ΔF/F). C) Photomicrograph of patched GCaMP6s expressing CRH neuron. Scale bar: 10 μm D) Relationship between total cumulative fluorescence and spike count per burst from a single representative neuron. E) Correlation r^2 and p values of total cumulative fluorescence and spike count in response to burst activity induced by 50 μM noradrenaline (blue, n=9) or 7.5 mM KCl (black, n=9).

Supplementary Information

Stress experience and hormone feedback tune distinct components of hypothalamic CRH neuron activity
Supplementary Figure 2

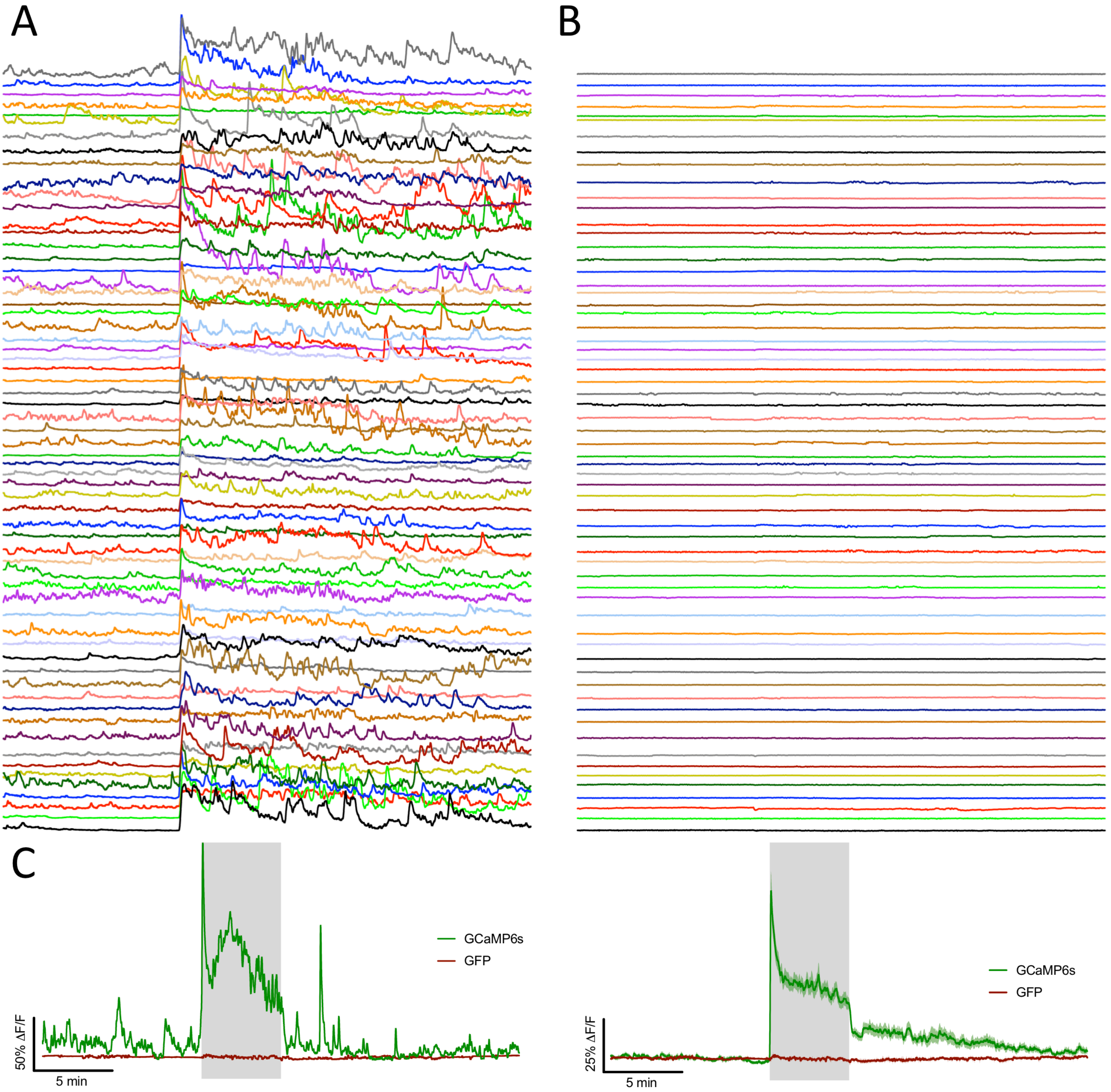

Supplementary Figure 2. Validation of CRH neuron photometry
A) Traces of control white noise responses and B) 405 nm reference signals in the same scale across all individual mice used in this paper n=64. C) Fluorescence changes in response to white noise stress in mice expressing GCaMP6s (green) or GFP (red) from representative individuals (left) and grouped mean (right; GFP n=3, GCaMP6s n=64).

Supplementary Information

Stress experience and hormone feedback tune distinct components of hypothalamic CRH neuron activity
Supplementary Figure 3

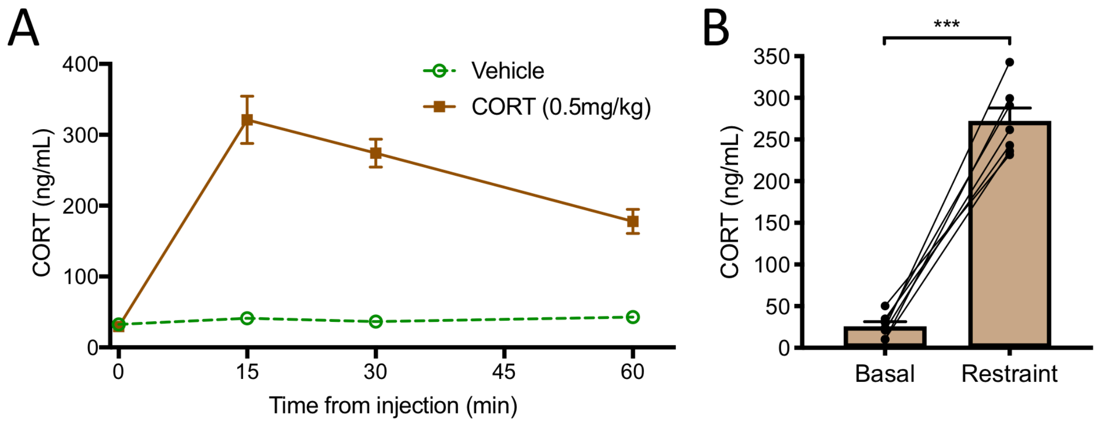

Supplementary Figure 3. Corticosterone injection
A) Changes in blood CORT concentrations following injections of either vehicle or CORT in metyrapone pre-treated mice. B) Elevations in blood CORT concentrations after a 10min restraint stress. Samples taken immediately before and 30min after stress onset. Paired t test, ***$p<0.001$. All data are represented as mean ± SEM.

Supplementary Information

Stress experience and hormone feedback tune distinct components of hypothalamic CRH neuron activity
Supplementary Figure 4

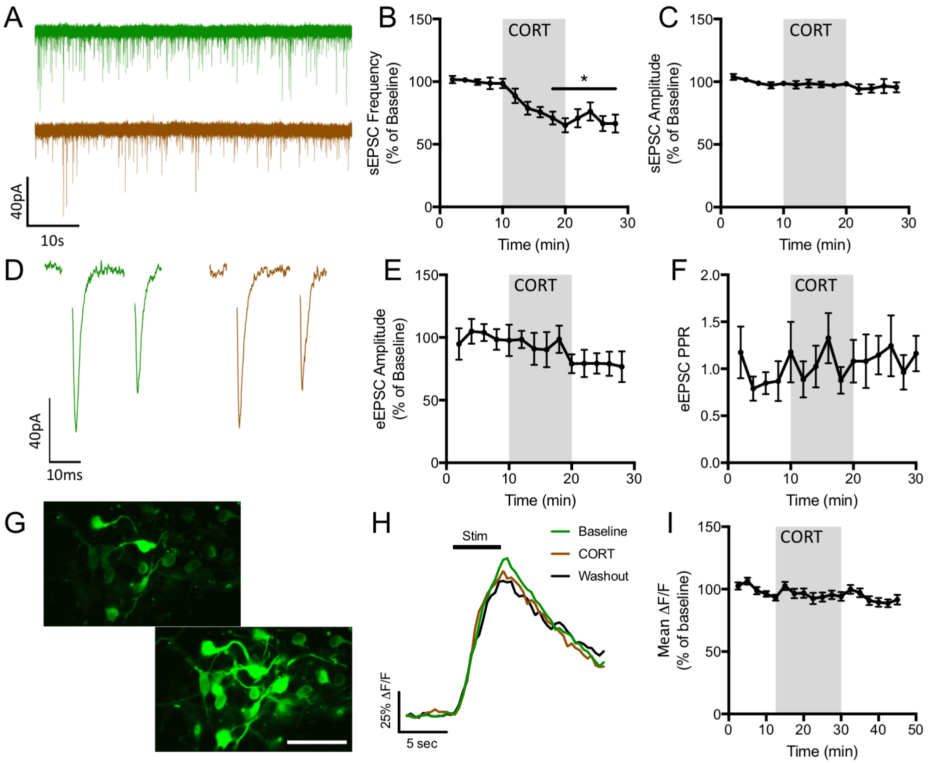

Supplementary Figure 4. CORT inhibits spontaneous glutamate release onto CRH neurons but does not affect evoked glutamate release nor electrically stimulated calcium signals.
A) Representative voltage-clamp traces of sEPSCs in before (green) and during bath application of CORT (1 µM, brown) in PVN CRH neurons. B) Changes in sEPSC frequency and C) Amplitude measured before, during, and after bath application of CORT (1 µM, n=9). *$p<0.05$, Friedman test. D) Representative voltage-clamp traces of eEPSC onto CRH neurons before (green) and during bath application of CORT (1 µM, brown). E) Changes in eEPSC P1 amplitude and F) PPR measured before, during, and after bath application of CORT (1 µM, n=8). G) Photomicrograph of GCaMP6s expressing CRH neurons before (top left) and during (bottom right) electrical stimulation (5 s stim, 10Hz, 100µA). Scale bar: 50 µm. H) Representative calcium fluorescence traces in response to electrical stimulation. I) Changes in evoked GCaMP6s fluorescence measured before, during and after bath application of CORT(1 µM, n=38 cells). All data are represented as mean ± SEM.

Supplementary Information

Stress experience and hormone feedback tune distinct components of hypothalamic CRH neuron activity
Supplementary Figure 5

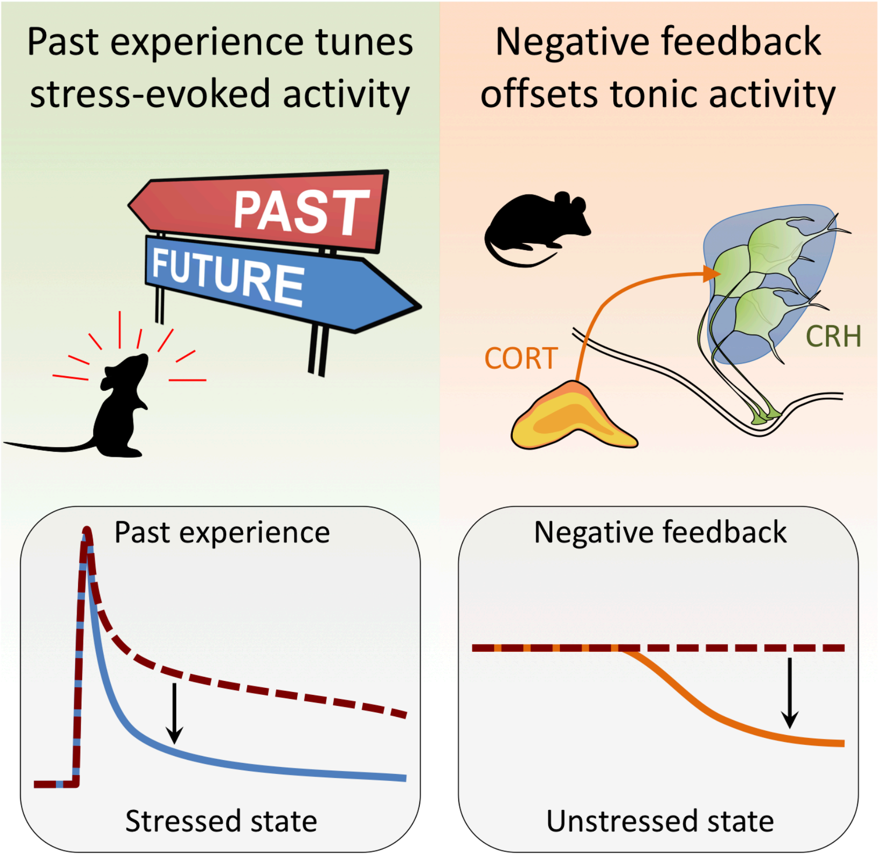

Supplementary Figure 5. Summary illustration of CRH neuron regulation by stress experience (left) and CORT feedback (right) on the different components of CRH neuron activity.

Supplementary Information

Stress experience and hormone feedback tune distinct components of CRH neuron activity
Supplementary Table 1

	# CRH neurons	# GCaMP neurons	# dual labelled neurons	% CRH with GCaMP	% GCaMP with CRH
	128.5 ± 6.3	84.3 ± 3.3	74 ± 2.8	58.1 ± 2.1	88.0 ± 1.8

Supplementary Table 1. Mean (±SEM) numbers of single- and dual-labeled CRH and GCaMP6s expressing neurons in the PVN (n=8).
