# Supplementary material for: Stress experience and hormone feedback tune distinct components of hypothalamic CRH neuron activity
Source: Nat Commun. 2019 Dec 13;10:5696. doi: 10.1038/s41467-019-13639-8 (PMC6911111; doi:10.1038/s41467-019-13639-8)
Supplement: Supplementary file 2 — Description of Additional Supplementary Files [file 41467_2019_13639_MOESM2_ESM.docx]

Description of Additional Supplementary Files

**Supplementary Movie 1.** CRH neuron activity during rest (tonic activity) and response to white noise stress (stress-evoked activity). Trace on right shows the real-time GCaMP6s fiber photometry signal aligned to the video (left). White noise stress is presented at 10 min (time as shown on video).
